# SHOVELCO: A novel adjustable-angle retractor for improved surgical field exposure in plastic and reconstructive surgery

**DOI:** 10.1016/j.jpra.2025.05.002

**Published:** 2025-05-14

**Authors:** Yuki Matsuoka, Natsuko Kakudo, Toshihito Mitsui, Masakatsu Hihara

**Affiliations:** Department of Plastic and Reconstructive Surgery, Kansai Medical University, Osaka, Japan

## Introduction

Achieving optimal exposure of the surgical field is essential for safe and effective procedures in plastic and reconstructive surgery. In surgeries such as tumor excision, flap elevation, and facial bone fracture repair, the operative field is often deep and obscured. Conventional retractors ^1^ are essential in such cases, but can be physically demanding for surgical assistants. Surgeons often demand more visibility, while assistants struggle with prolonged and forceful traction, leading to mutual frustration. To address these challenges, we developed *SHOVELCO*, an innovative retractor equipped with an adjustable-angle mechanism. This device was designed to optimize surgical exposure while minimizing assistant fatigue.

## Materials and methods

### Design features of the SHOVELCO retractor

The *SHOVELCO* retractor incorporates three key structural features that distinguish it from conventional instruments:

1. Adjustable tip angle:
  A rotating dial located near the handle allows the user to modify the tip angle across a range of approximately 55° to 125°, enabling customized positioning depending on the depth and orientation of the surgical field (Figure 1a, b).

**Figure 1:**
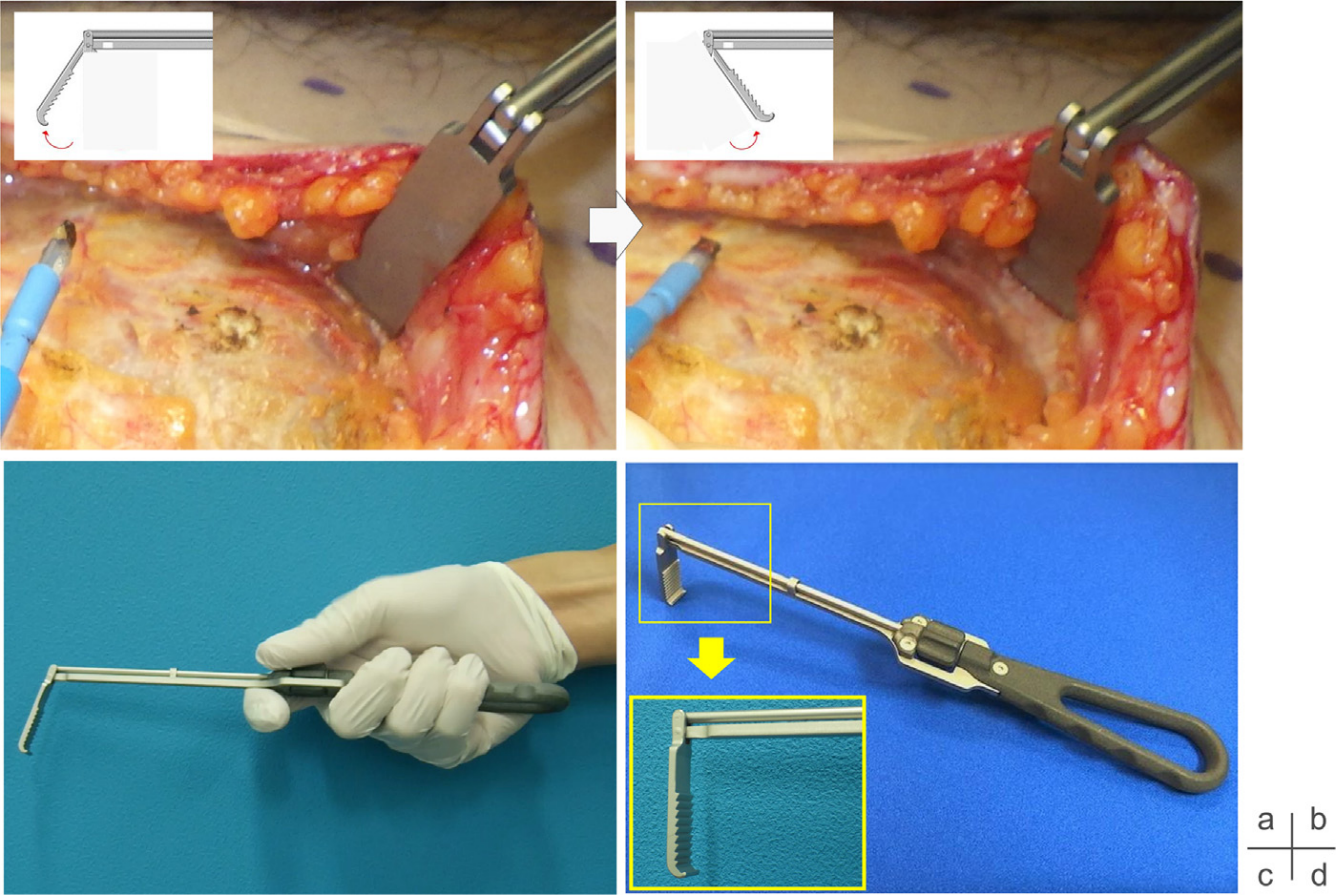
The tip angle can be adjusted from 125° (a) to 55° (b). The dial can be operated during retraction for real-time modifications (c). The tip features grooves on the deep side for improved grip, while the superficial side remains smooth to prevent soft tissue damage (d).
2. Intraoperative angle modulation:
  The angle can be adjusted even while the instrument is under tension. This allows for real-time optimization of the surgical view without interrupting the procedure (Figure 1c). The mechanism is designed to ensure smooth operation without requiring disengagement or repositioning.
3. Functional tip geometry:
  The tip features finely engineered serrations that grip soft tissues effectively, reducing slippage during retraction. To avoid unnecessary trauma to the skin, the contact surface is smoothed and rounded (Figure 1d). This balance of grip and protection is particularly advantageous in cases involving fatty or slippery tissues.

### Locking mechanism

To ensure secure angle retention during use, the SHOVELCO retractor employs a screw-based locking mechanism integrated into the rotary knob. When the knob is turned, it moves an internal shaft that adjusts the angle of the tip via a pivot. The friction generated between the threaded surfaces provides mechanical resistance sufficient to prevent slippage, even under surgical loads. Additionally, a spring-loaded system supports the knob, further minimizing unintended rotation. A schematic diagram of this mechanism is shown in supramental supplemental Figure.

### Clinical application and case examples

**Figure 2:**
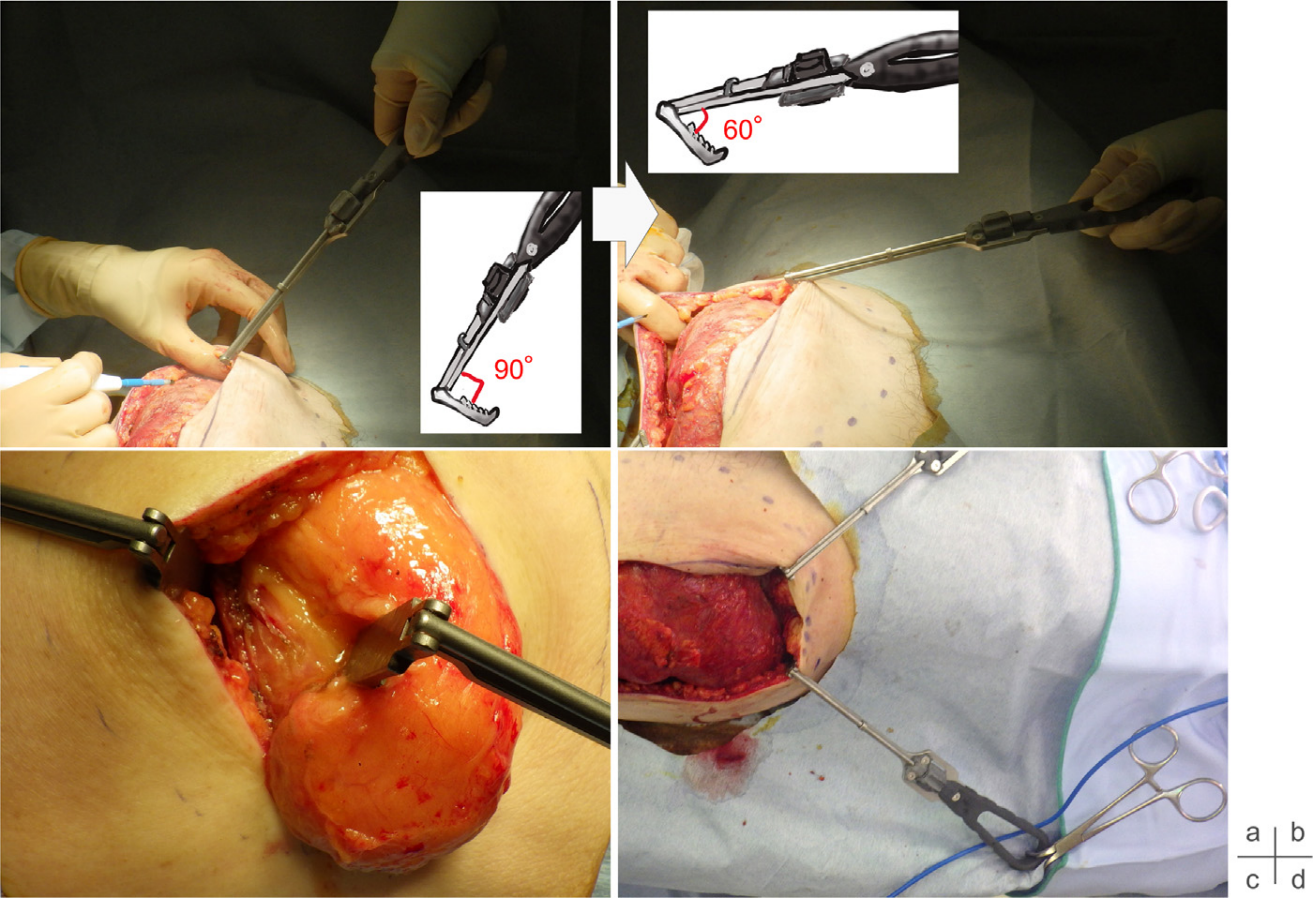
Adjusting the tip angle from 90° (a) to 60° (b) enables retraction in a less strenuous direction, improving surgical field exposure. The textured tip allows for stable lipoma retraction (c) and can function as a self-retaining retractor (d).
• Improved control over slippery tissues:
  The serrated design of the tip proves especially useful when dealing with lipomas and other slippery tissue masses. It enables effective stabilization with minimal effort, unlike conventional smooth retractors which often require repeated repositioning (Figure 2c).
• Self-retaining functionality:
  When the tip is adjusted to a sharper angle, the retractor can be secured using standard towel clamps or forceps, functioning as a self-retaining device. This eliminates the need for continuous manual traction, further decreasing assistant workload and enhancing procedural efficiency (Figure 2d).

We applied *SHOVELCO* in a variety of procedures, including subcutaneous tumor resections, facial bone fracture surgeries, and reconstructive flap surgeries. Across these applications, the following clinical benefits were observed:

• Reduced assistant fatigue:
  By adjusting the tip to redirect traction horizontally rather than vertically, the assistant can apply less force while still achieving optimal field exposure. This ergonomic improvement significantly reduces muscle strain and improves procedural comfort for the assistant (Figure 2a and b).

## Discussion

The *SHOVELCO* retractor addresses several longstanding issues encountered during surgical field exposure. Its ergonomic and adaptive design contributes to improved collaboration between the surgeon and assistant. Moreover, it enables even junior assistants or residents to achieve stable retraction, enhancing the learning environment. The locking mechanism offers strong, reliable fixation via internal friction and a spring-supported dial.

Nevertheless, there are limitations. The serrated tip, while effective for grip, requires cautious handling around delicate structures to avoid unintended trauma. Additionally, the current version is available in only one size (10 mm width × 34 mm length), which may limit its utility in particularly deep or wide surgical fields. Future improvements may include variable tip designs and integrated lighting.

## Conclusion

*SHOVELCO* is a novel retractor that enables efficient and adjustable surgical field exposure. Its angle-adjustable mechanism and functional tip design significantly reduce assistant workload while maintaining excellent visualization for the surgeon. The device is especially useful in subcutaneous tumor excisions, facial fracture repairs, and reconstructive procedures, where stable exposure is critical. We believe *SHOVELCO* represents a practical and innovative solution that can be beneficial even in settings with limited assistant availability or for training purposes.

## Ethics

Not applicable.

## Declaration of generative AI and AI-assisted technologies in the writing process

During the preparation of this work, the author(s) used ChatGPT in order to assist with proofreading and improving the clarity of the manuscript. After using this tool, the author(s) reviewed and edited the content as needed and take(s) full responsibility for the content of the publication.

## Funding

No specific funding was received for this study.

## Supplementary material

Supplementary Figure**.** Schematic illustration of the locking mechanism used in the SHOVELCO retractor. The angle of the tip is controlled by a rotary knob which actuates a shaft through an internal screw. The friction between the screw and shaft prevents unintentional rotation under load. A spring mechanism enables secure angle retention without external fixation.

## Declaration of competing interest

The authors declare no conflicts of interest.

## Appendix Supplementary materials

Image, image 1
