# Supplementary material for: SHOVELCO: A novel adjustable-angle retractor for improved surgical field exposure in plastic and reconstructive surgery
Source: JPRAS Open. 2025 May 14;45:109–12. doi: 10.1016/j.jpra.2025.05.002 (PMC12197876; doi:10.1016/j.jpra.2025.05.002)
Supplement: Supplementary file 1 [file mmc1.docx]

Schematic illustration of the locking mechanism used in the Shovelco retractor. The angle of the tip is controlled by a rotary knob which actuates a shaft through an internal screw. The friction between the screw and shaft prevents unintentional rotation under load. A spring mechanism enables secure angle retention without external fixation.
